# Characterization of the complete chloroplast genome sequence of *Pennisetum glaucum* and its phylogenetic implications

**DOI:** 10.1080/23802359.2019.1668312

**Published:** 2019-10-24

**Authors:** Jin Xu, Yun Song, Xiaoyan Jing, Mingfu Li

**Affiliations:** Chinese Academy of Inspection and Quarantine, Beijing, China

**Keywords:** Chloroplast genome, *Pennisetum glaucum*, phylogenetic analysis

## Abstract

*Pennisetum glaucum* is a high nutritive-value summer-annual forage crop, popular among livestock producers for grazing, silage, hay, and green chop. In this study, the complete chloroplast genome of the *P. glaucum* was assembled from the whole genome Illumine sequencing data. The size of the *P. glaucum* chloroplast genome is 138,119 bp, including a large single-copy region (81,034 bp), a small single-copy region (12,409 bp), and a pair of inverted repeats regions (22,338 bp). The overall GC content of the *P. glaucum* chloroplast genome was 38.6%. The chloroplast genome of *P. glaucum* encodes 110 different genes, including 76 protein-coding genes, 30 transfer RNAs (tRNA), and four ribosomal RNAs (rRNA). Phylogenetic analysis confirmed a close relationship of *P. glaucum* with species in the Panicoideae subfamily of the Poaceae family.

*Pennisetum glaucum* is a major food and fodder crop for farmers living on marginal agricultural lands in the arid and semi-arid tropics of Africa and Asia (Gupta et al. 2018). *Pennisetum glaucum* can also be utilized as emergency forage that regularly performs well as an economical 1-year forage crop option (Ed Jennings et al. 2017). However, only a few genomic resources have been explored. To facilitate its genetic research and contribute to its utilization, in this study, the complete genome of the *P. glaucum* was assembled from the whole genome illumine sequencing data. Phylogenetic analysis was conducted, which will be useful for further studies on its chloroplast genetic engineering.

The sample was collected from Tangshan, Hebei province, China (39°28′11″E, 118°32′43″N), and was deposited at Chinese Academy of Inspection and Quarantine (Voucher No. 2019099PG01). Total genomic DNA was extracted following the method of Jinlu et al. (2013). Genomic DNA was sequenced using Illumina Hiseq 4000 PE150 platform in Beijing Biocode Biotech Co., Ltd (Beijing, China). Approximately 12,845,315 reads were generated from the sequencing library. The reads were qualitatively assessed and assembled with SPAdes 3.6.1 (Bankevich et al. 2012). The chloroplast contigs were selected and sorted by comparison with reported chloroplast genome sequence (Altschul et al. 1997). The selected contigs were assembled using Sequencher 4.10 and then merged and gap-filled by a series of read mapping using Geneious 8.1 (Kearse et al. 2012). The annotation was performed with Plann (Huang and Cronk 2015). Finally, the whole chloroplast genome map was generated using Organellar Genome DRAW (https://chlorobox.mpimp-golm.mpg.de/OGDraw.html) (Lohse et al. 2013). The sequence of *P. glaucum* complete chloroplast genome was submitted to Genbank with the accession number MN180104.

The complete chloroplast genome of *P. glaucum* is 138,119 bp in length. The genome’s circular, quadripartite structure is composed of SSC with the length of 12,409 bp and LSC with the length of 81,034 bp, separated by a pair of IR element (IRA and IRB) with the length of 22,338 bp. The GC content of its chloroplast DNA is 38.6% while the corresponding values of the LSC, SSC, and IR regions are 36.5%, 33.0%, and 44.0% separately. It encodes a total of 110 genes (76 protein-coding, 30 tRNA, and four tRNA). Intron-exon structure analysis indicated that 17 genes contained intron, in which two of them (*ycf3* and *clpP*) had two introns while the others had one intron. *rps12* is a special trans-splicing gene which 5'-terminal exon is located in LSC and 3'-terminal exon is located in IR region.

Phylogenetic analysis of *P. glaucum* with other taxa was performed using a maximum-likelihood (ML) method with whole chloroplast genome sequences. ML was used in RAxML8.0 (Stamatakis 2014). Relative clade support was estimated by ML bootstrap analysis of 1000 replicates of heuristic searches with the GTR + G model. The results indicated that *P. glaucum* formed a group with species in the Panicoideae subfamily of the Poaceae family (Figure 1). The genome information reported here could be further applied for evolution and population genetics, molecular studies in this plant species and family.

**Figure 1.**
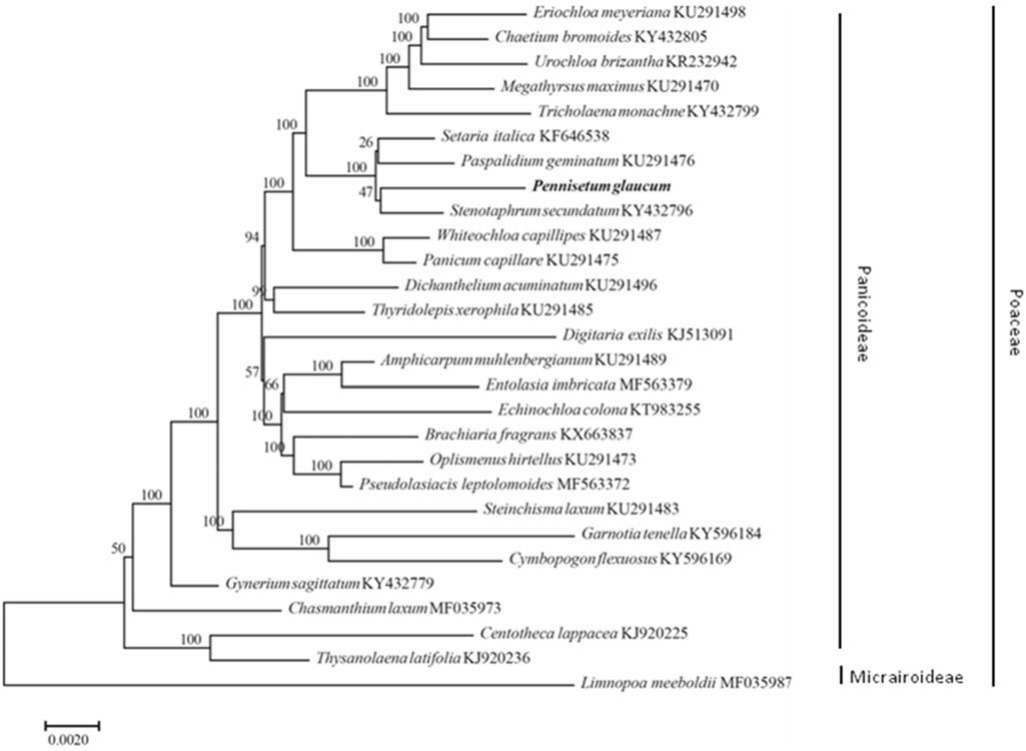
Phylogeny of *Pennisetum glaucum* and other 27 species belonging to the Gramineae based on the complete chloroplast genome sequences using the maximum-likelihood method.

## Data Availability

The chloroplast genome sequence of the *P. glaucum* was submitted to Genebank of NCBI. The accession number from Genebank is MN180104.
